# Comprehensive genomic profiling of Finnish lung adenocarcinoma cohort reveals high clinical actionability and *SMARCA4* altered tumors with variable histology and poor prognosis

**DOI:** 10.1016/j.neo.2022.100832

**Published:** 2022-08-11

**Authors:** Eva-Maria Talvitie, Lassi Liljeroos, Heikki Vilhonen, Katri Orte, Ilmo Leivo, Markku Kallajoki, Pekka Taimen

**Affiliations:** a Department of Genomics, Turku University Hospital, Kiinamyllynkatu 10, 20520 Turku, Finland; b Roche Oy, Revontulenpuisto 2C, 02100 Espoo, Finland; c University of Turku, Department of Pulmonary Diseases and Clinical Allergology and Division of Medicine, Department of Pulmonary Diseases, Turku University Hospital, Hämeentie 11, 20521 Turku, Finland; d Department of Pathology, Turku University Hospital, Kiinamyllynkatu 10, 20520 Turku, Finland; e Institute of Biomedicine and FICAN West Cancer Centre, University of Turku, Kiinamyllynkatu 10, 20520 Turku, Finland

**Keywords:** Lung adenocarcinoma, Mutational landscape, Never-smoker, SMARCA4, TMB, Tumor mutation burden

## Abstract

**Introduction:**

Lung adenocarcinoma is the most common type of lung cancer and typically carries a high number of mutations. However, the genetic background of the tumors varies according to patients’ ethnic background and smoking status. Little data is available on the mutational landscape and the frequency of actionable genomic alterations in lung adenocarcinoma in the Finnish population.

**Materials and methods:**

We evaluated the gene alteration frequencies of 135 stage I–IV lung adenocarcinomas operated at Turku University Hospital between 2004 and 2017 with a large commercial comprehensive genomic profiling panel. Additionally, we correlated the alterations in selected genes with disease outcomes in 115 stage I–III patients with comprehensive follow-up data. The genomic alterations in a sub-cohort of 30 never-smokers were assessed separately.

**Results:**

Seventy percent of patients in the overall cohort and 77% in the never-smoker sub-cohort harbored an alteration or a genomic signature targetable by FDA and/or EMA approved drug for non-small cell carcinoma, respectively. In multivariable analysis for disease-specific survival, any alteration in *SMARCA4* (DSS; HR 3.911, 95%CI 1.561–9.795, *P*=0.004) exhibited independent prognostic significance along with stage, tumor mutation burden, and predominant histological subtypes.

**Conclusions:**

Over two thirds of our overall cohort, and especially never-smokers had an actionable genomic alteration or signature. *SMARCA4* alterations, detected in 7.4% of the tumors, independently predicted a shortened overall and disease-specific survival regardless of the alteration type. Most *SMARCA4* alterations in our cohort were missense mutations associated with differentiated predominant histological subtypes and immunohistochemical SMARCA4/BRG1 and TTF-1 positive status.

## Introduction

Lung cancer is the leading cause of cancer-related mortality worldwide [1]. Most lung cancers are non-small cell lung cancers (NSCLC), and adenocarcinoma is the most common subtype with an increasing trend in relative incidence [2]. Adenocarcinomas harbor targetable genomic alterations in *ALK, BRAF, EGFR,* and *ROS1* genes, and novel treatment options such as *KRAS* Gly12Cys*, MET, NTRK,* and *RET* inhibitors, and possibly *ERBB2* guided therapies are broadening the clinical repertoire [3], [4], [5]. In addition to guiding the treatment of individual cancers, genomic alterations may indicate resistance to targeted therapies [6] and immunotherapy [7], as well as conventional chemotherapy [8]. As the mutational landscape varies by ethnic background, knowledge of the local mutational frequencies will help allocate resources to detect clinically significant alterations in cases where broad genomic profiling is not routinely used. Finns differ genetically from other European populations, and there is little previous data on the mutational landscape of Finnish lung adenocarcinoma patients [9].

Most of the lung adenocarcinoma patients in the Western population are current or ex-smokers, but the proportion of never-smokers is expected to increase as smoking declines. Adenocarcinomas in never-smokers are genetically different from those detected among smokers as the latter group has a significantly higher number of individual mutations and different mutation types [10], [11], [12]. Tumors with *EGFR* mutations are common in never-smokers, occurring in 27.4–66.7% of European [13], [14], [15], [16], [17], [18] and 60–78% of East Asian [19] patients without a smoking history. Besides *EGFR*, never-smoking status is associated with oncogenic gene fusions involving *ALK1, ROS1*, and *RET*. The genomic data on European never-smokers is still relatively scarce, and to our knowledge, there is no published genomic data about adenocarcinomas among never-smokers in the Finnish population.

Here we mapped the mutational landscape of lung adenocarcinomas in a single-center patient cohort via a large commercial comprehensive genomic profiling panel, emphasizing currently recognized actionable alterations and alterations in never-smokers.

## Materials and methods

Our retrospective cohort consisted of 135 patients with stage I–IV primary invasive lung adenocarcinoma operated in Turku University Hospital in 2004–2017 with curative intent. Two patients had received neoadjuvant chemotherapy, while none had received radiotherapy. Three patients underwent immunotherapy in 2017–2018. We traced the smoking status and other clinicopathological characteristics from the electronic patient records. A never-smoker was defined as a person who self-reportedly had consumed less than 100 cigarettes in their lifetime. We acquired the day and cause of death through Statistics Finland, with the last follow-up day on 31.12.2018. We excluded patients from survival analysis based on the following criteria: incomplete clinical follow-up data, death within 30 postoperative days, macroscopic (R2) residual disease, and immunotherapy during the follow-up period. The collection of clinical patient data was approved by the administration of the Hospital District of Southwest Finland (T150/16), and the use of tissue material was approved by the Scientific Steering Committee of Auria Biobank (AB14-8689 and AB20-9755). We conducted the study in collaboration with Auria Biobank and Roche Oy (Espoo, Finland).

## Genomic characterization

FoundationOne (Foundation Medicine, Inc., Cambridge, MA, USA) comprehensive genomic profiling, described and validated by Frampton et al. [20], was performed on ten 5 µm thick formalin-fixed paraffin-embedded tissue sections on charged and unbaked slides. We omitted tumors with an insufficient number of non-necrotic tumor cells and those limited to a single tumor block per patient. FoundationOne provided the genomic data classified as short variant mutations (single nucleotide variants (SNVs) and indels of 1–40 base pairs), copy number alterations (amplifications and losses), and rearrangements. These were further categorized as either known pathogenic, likely pathogenic, or variants of uncertain significance (VUS). The FoundationOne panel version used in our study covered the exons of 315 genes and selected intronic regions [20].

## Immunohistochemical staining

Immunohistochemical SMARCA4/BRG1 stainings were performed at Fimlab Laboratories (Tampere, Finland) using an in-house validated protocol. In brief, tissue sections of 4 µm thickness were cut from the same paraffin blocks used for sequencing, deparaffinized with xylene, and rehydrated in a series of ethanol. Antigen retrieval was performed with Cell Conditioning 1 (CC1) solution and heat-induced epitope retrieval (HIER). Staining was performed on Ventana BenchMark ULTRA staining instrument using rabbit monoclonal SMARCA4/BRG1 antibody (1:100, clone EPNCIR111A, Abcam, Cambridge, UK), and the sections were counterstained with hematoxylin. Lymphatic tissue on the same slide was used as a positive external control, and intratumoral lymphocytes were used as internal positive controls.

## Statistical analyses

The clinicopathological and genomic data were correlated with the χ2 test and Fischer´s exact test. Overall survival (OS) and disease-specific (DSS) survival were estimated using the Kaplan-Meier method and the log-rank test. The genomic data were adjusted for clinical parameters with multivariable Cox regression analysis. P-values less than 0.05 were considered statistically significant. Statistical analyses were performed with SPSS (IBM, version 28, 2021). The waterfall plots were created with R (R Project, version 3.6.1, 2020) GenVisR package and modified with GIMP.

## Results

### Patient characteristics

A total of 135 patients with surgically treated primary lung adenocarcinoma and available FoundationOne genomic data were included in the study. All the patients were of Finnish European descent. After applying the exclusion criteria, 115 patients remained in the survival analyses. The never-smoker sub-cohort consisted of 30 patients (70% women). The mean follow-up period was 56.1 months (range 7.0–173.5 months). The clinical characteristics of the cohort are summarized in Table 1.

**Table 1:** Clinical characteristics of the cohort.

		No. of patients (%) (*n*=135)	No. of patients in survival analyses (%) (*n*=115)
**Mean age at operation**		66	66
**Sex**			
	Female	65 (48.1)	55 (47.8)
	Male	70 (51.9)	60 (52.2)
**Smoking**			
	No	30 (22.2)	29 (25.2)
	Yes	104 (77.0)	86 (74.8)
	Unknown	1 (0.7)	0
**Type of surgery**			
	Sublobar resection	7 (5.2)	4 (3.5)
	Lobectomy	89 (65.9)	78 (67.8)
	Bilobectomy	34 (25.2)	31 (27.0)
	Pneumonectomy	4 (3.0)	2 (1.7)
	Unknown	1 (0.7)	0
**Stage (TNM8)**			
	I	71 (52.6)	68 (59.1)
	II	27 (20.0)	24 (20.9)
	III	31 (23.0)	23 (20.0)
	IV	5 (3.7)	0
	Unknown	1 (0.7)	0
**Residual disease**			
	No	112 (83.0)	108 (93.9)
	Microscopic	7 (5.2)	7 (6.1)
	Macroscopic	14 (10.4)	0
	Unknown	2 (1.5)	0

### Mutational landscape of tumors

The tumors harbored 2682 individual non-synonymous genomic alterations (2366 SNVs and small indels, 281 copy number alterations, and 35 rearrangements), ranging from four to 99 alterations per tumor. The average median exon coverage was 571. Median tumor mutational burden (TMB) was 7.02 (range 0.0–88.6), and 49 patients had TMB of at least ten mutations/MB. Microsatellite instability was present in one tumor, co-occurring with high TMB (35.1 mutations/MB).

**Fig. 1:**
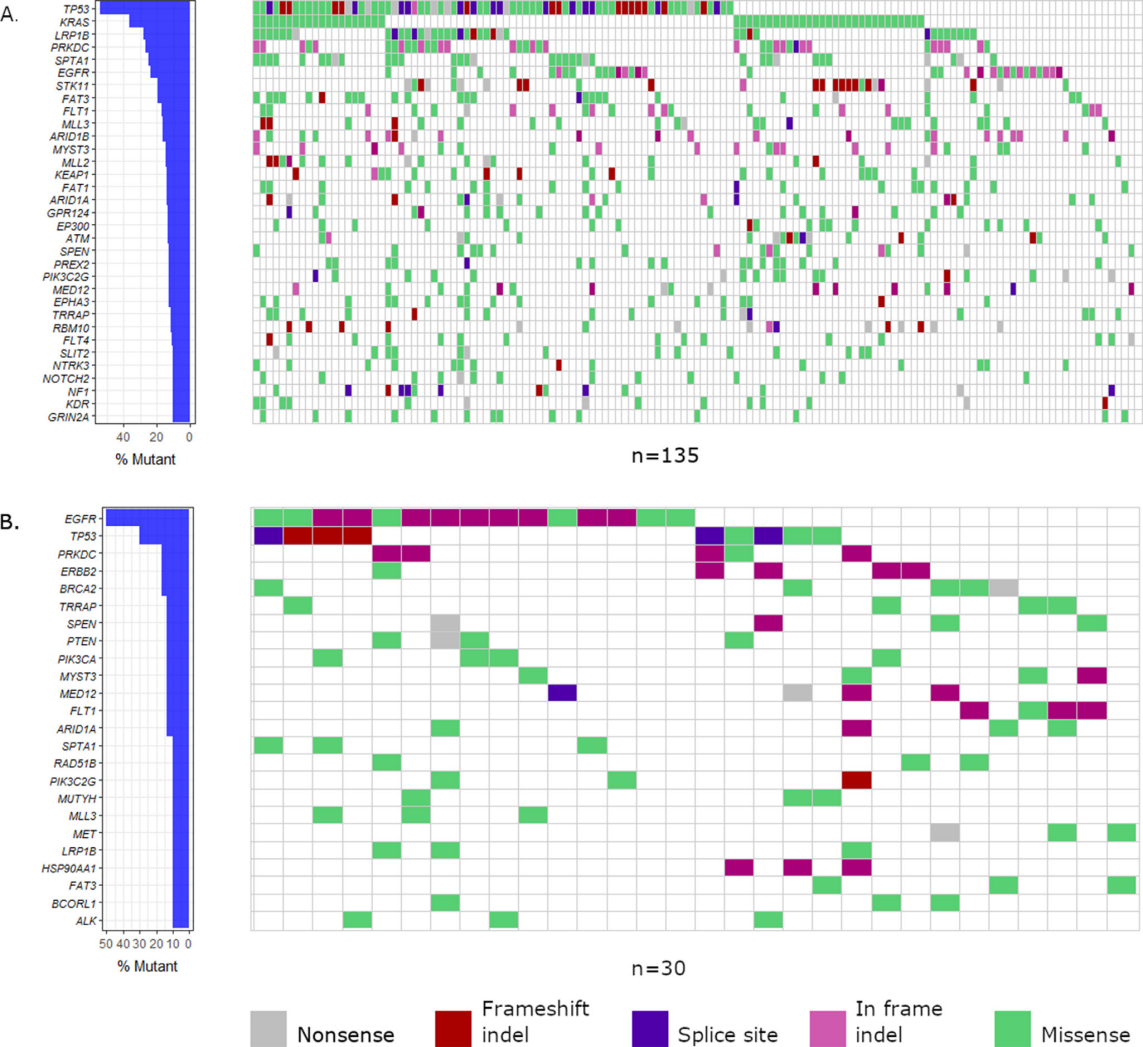
A waterfall plot of short variant mutation frequencies in genes with mutations in at least 10% of patients. Mutations in A) the whole cohort and B) the never-smoking sub-cohort are shown.

*Overall,* TP53 was the most commonly altered gene, with 54.8% of tumors having TP53 alterations of any kind. One or more alterations in *KRAS* were detected in 37.8% of the tumors, followed by *LRP1B, SPTA1*, and *PRKDC* (28.1% each), *EGFR* (23.7%), and *STK11* (20.0%). *KEAP1* was mutated in 14.1% and *NFE2L2* in 5.9% of the tumors. The genes with mutations in at least 10% of patients in the overall cohort are presented in Fig. 1A. The frequencies of individual genomic alterations of all the genes included in the panel are reported in Supplementary Table S1 and all the alterations in selected genes relevant to lung adenocarcinoma in Supplementary Table S2.

All tumors in the cohort harbored at least one known or likely pathogenic alteration. *TP53* had the highest number of pathogenic or likely pathogenic variants, with 82 alterations in 73 individual tumors. The subsequently most affected genes were *KRAS* (54 pathogenic or likely pathogenic alterations in 51 tumors), *EGFR* (36 alterations in 29 tumors), *STK11* (23 alterations in 22 tumors), and *RBM10* (14 alterations in 14 tumors).

### Established genomic biomarkers per European Society for Medical Oncology guidelines

At the time of writing this article, the European Society for Medical Oncology (ESMO) guidelines recommended the molecular subtyping of *EGFR, ALK, ROS1, BRAF*, and *NTRK* as predictive biomarkers for first-line targeted therapies in metastatic non-small cell carcinoma [21]. Over a third of our patients (31.9%) harbored a pathogenic or likely pathogenic alteration in these genes, mutually exclusively. A total of twenty-nine patients (21.5%) had one or more alterations in *EGFR*, nine patients (6.7%) in *BRAF*, three patients (2.2%) in *RET*, and one patient each (0.7%) in *ALK* and *ROS1.* There were no *NTRK* fusions.

*EGFR* exon 19 deletions were identified in 13 patients (44.8% of patients with pathological or likely pathological *EGFR* alterations) and Leu858Arg *EGFR* mutation in eight patients (27.6%). Two patients had tumors with compound Gly719Ala and Ser768Ile *EGFR* mutations.

As our patients were treatment naïve at the time of their surgery, no acquired *EGFR* tyrosine kinase inhibitor (TKI) resistance mutations were present, and neither were primary resistance mutations observed. *EGFR*-*TP53* co-alterations, shown to associate with shorter progression-free survival following 1^st^ or 2^nd^ generation *EGFR* TKI treatment [22], were seen in 16 tumors. Four patients harbored *EGFR* exon 20 in-frame insertion, conferring resistance to *EGFR* TKIs but sensitizing to amivantamab. *EGFR* was amplified in four tumors, with three having a concurrent activating *EGFR* mutation and one having an exon 20 insertion, potentially modifying *EGFR* TKI treatment response.

Five patients had a *BRAF* Gly469Val mutation, while four patients had other non-Val600 mutations, and one patient had a compound Gly469Val and Ser605Cys mutation. There were no Val600Glu mutations. A previously reported *RET* fusion was present in three patients, with two having *RET*/*KIF5B* fusions and one having a *RET*/*CCDC6* fusion. One patient had a previously unreported *RET/PTPLA* fusion with a concurrent *KRAS* Gly12Val mutation. There was one case of *ALK/EML4* and *ROS1/CD74* fusion each (0.7%).

In conclusion, 24 patients had an *EGFR* mutation with an indication for *EGFR* TKIs (exon 19 deletions, Leu858Arg mutation, and Gly719X mutations), four patients had an exon 20 insertion for which amivantamab is recommended, and eight patients had *EGFR* alterations predicting a decreased response to *EGFR* TKIs (*EGFR* exon 20 insertion, *EGFR* amplification). Five tumors harbored fusion genes with available targeted treatment (*ALK, ROS1, RET*) (Table 2).

**Table 2:** Actionable and potentially actionable mutations and genomic signatures across the cohort (n=135). TKI=Tyrosine kinase inhibitor, FDA=U.S. Food and Drug Administration, EMA=European Medicines Agency, MSI-H=microsatellite instability.

Biomarker	Alteration	N:o ofpatients (%)	Never smokers	Therapy	Approval or evidence
***EGFR***	Exon 19 deletion	13 (9.6)	7	1-3. generation *EGFR* TKI:s	EMA, FDA
	Leu858Arg	8 (5.9)	5		
	Gly719Ala + Ser768Ile	2 (1.5)	0	Osimertinib * * Cai Y, Wang Y, Sun J, et al. *J Int Med Res*. 2020	
	Gly719Cys	1 (0.7)	0		
	Exon 20 insertion	4 (3.0)	3	Amivantamab-vmjw	
	Amplification	4 (3.0)	2		
***ERBB2***	Exon 20 insertions	4 (3.0)	2	Ado-trastuzumab emtansine, fam-trastuzumab deruxtecan-nxki	NCCN guidelines,evidence level 2A
	Amplification	2 (1.5)	0	Trastuzumab+paclitaxel?	

***MET***	Exon 14 splice site mutations	3 (2.2)	0	Crizotinib, capmatinib, tepotinib	FDA
	D1010H	1 (0.7)	1		
	Y1003X	2 (1.5)	1		
***KRAS***	Gly12Cys	20 (14.8)	0	Sotorasib, adagrasib **	EMA, FDA**
***ROS1***	Fusion	1 (0.7)	1	Crizotinib, entrectinib	EMA, FDA
***RET***	Fusion	3 (2.2)	3	Pralsetinib, selpercatinib	EMA, FDA
***ALK***	Fusion	1 (0.7)	1	1-2. generation *ALK* inhibitors	EMA, FDA
***BRCA1/2***		4 (3.0)	0	PARP inhibitors	Approved in other solid tumor types
**MSI-H**		1 (0.7)	0	Pembrolizumab	FDA
**TMB ≥ 10**		49	1	Pembrolizumab	FDA

### Emerging genomic biomarkers

The genomic biomarkers not yet recommended by ESMO in routine practice but already present in the NCCN, CAP/IASCL/AMP, and ASCO guidelines include *KRAS, ERBB2*, and *MET* [23]. Additionally, FDA has accepted tumor mutation burden (TMB) as a biomarker for immunotherapy with pembrolizumab.

In *KRAS*, the most common genomic alteration was Gly12Cys (20 patients, 35.1% of patients with any *KRAS* alterations). Other pathogenic and likely pathogenic *KRAS* alterations, to which presently no targeted treatment is available, were present in 37 patients (Supplementary Table S2).

The most common *ERBB2* alteration was an exon 20 insertion, present in four patients. Two patients had an *ERBB2* amplification. One patient had an activating exon 8 mutation Ser310Phe, and one patient had an activating exon 19 Asp769His mutation. In *MET*, six exon 14 alterations were identified, including three exon 14 skipping splice site mutations, two Tyr1003X mutations, and one Asp1010His mutation. *MET* amplification, indicating resistance to *EGFR* TKIs and sensitivity to small molecule *MET* inhibitors, was present in three tumors.

In conclusion, 26 patients had a variant (20 *KRAS* Gly12Cys and six *MET* exon 14 alterations) targetable with drugs already accepted by the U.S. Food and Drug Administration (FDA) and the European Medicines Agency (EMA). Additionally, four *ERBB2* exon 20 insertions and one exon 19 SNV, recommended to be treated with trastuzumab-drug conjugates in the NCCN guidelines, were present. Furthermore, three cases of *MET* amplification were found.

### Additional alterations in homologous recombination repair genes, and co-alterations in key genes relevant to cancer

Defects in genes involved in homologous recombination repair (HRR) are being trialed as histology-agnostic biomarkers for PARP inhibitors in various solid malignancies, either alone or in combination with chemotherapy, targeted therapy, or immunotherapy. In non-small cell lung cancer, the prevalence of HRR gene deficiencies has been reported to be approximately 15% [24]. In our cohort, 18 individual tumors (13.3%) had known or likely pathogenic alterations in the following HRR associated genes: *ATM* in eleven patients, *BRCA1/2* in four patients, *BRIP1* in one patient, *CHEK2* in three patients, *RAD50* and *RAD54L* in two patients each, and one alteration each in *FANCM, BARD1, BRIP1*, and *MRE11A*. We were unable to confirm whether these variants were germline or somatic in origin.

Co-alterations involving *KRAS, TP53, STK11*, and *CDKN2A/B* were present in 33% of the whole cohort: *KRAS*-*TP53* in 16.3%, *KRAS*-*STK11* in 8%, *TP53*-*STK11* in 9%, and *KRAS*-*CDKN2A/B* loss in 4% of the tumors. *KRAS*-*STK11* -alterations have been associated with a poor response to immune checkpoint inhibitor therapy [7]. *MDM2* amplification, suggested being linked with hyperprogression during immunotherapy in a small series of various malignancies [25,26], was present in 7% of the patients. Two patients with a co-occurring *EGFR*-*TP53*-*RB1* alteration, predisposing to transformation to small cell carcinoma or squamous cell carcinoma following *EGFR* TKI treatment [27,28], were observed.

### Clinical actionability in the cohort

In the overall cohort, 59 patients (27 never-smokers) had a genomic alteration, and 49 patients a genomic signature (TMB ≥ 10, MSI-H or both, one never-smoker) for which an FDA and/or EMA approved NSCLC-indicated targeted treatment is currently available (Table 2, March 2022). Of these patients with targetable genomic alterations, 13 (22%) also had a TMB ≥ 10. Thus, overall 95 of our patients (70.4%) had alteration(s) or a genomic signature with an approved targeted drug available. Furthermore, additional alterations with a treatment option in other solid malignancies were detected in nine tumors (four *ERBB2* exon 20 insertions, one *ERBB2* Ser310Phe SNV, and four *BRCA1/2* alterations). All the targetable or potentially targetable alterations were mutually exclusive except for one patient with a *KRAS* Gly12Cys and a concurrent *BRCA2* variant.

### The effect of smoking on genomic alterations

The mean age at the time of diagnosis was 71.5 years for smokers, and 64.6 years for never-smokers. Never-smokers had 332 individual genomic alterations (260 short-variants, 63 copy number alterations, and nine rearrangements; 5–19 alterations per patient).

**Fig. 2:**
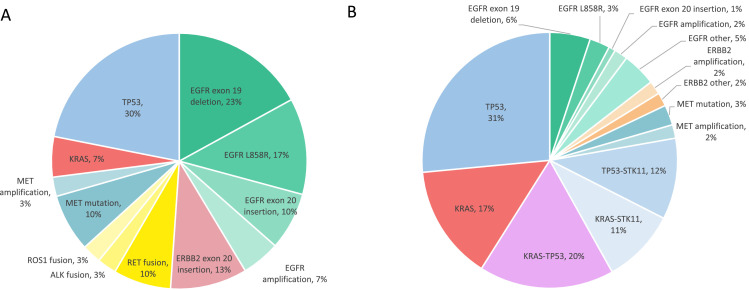
The genomic alterations with current clinical relevance in lung adenocarcinoma. Frequencies of alterations in A) never-smoking patients and B) in patients with a smoking history.

The most frequently altered genes in never-smokers were *EGFR* (50.0% of patients harboring one or more alterations), *TP53* (30.0%), *FRS2* and *MDM2* (26.7% each), *CDKN2A* and *CDKN2B* (7.9% each), *BRCA2, ERBB2, PRKDC, PIK3CA, PTEN, SPEN* and *SPTA* (16.7% each), while alterations in other genes were found in four patients (13.3%) or less. The pathogenic *ERBB2* variant Ala775_Gly776insYVMA (four patients) and *PTEN* variant of unknown significance Asp268Glu (three patients) were found exclusively in never-smokers. Other variants detected only in never-smokers presented in only one or two patients. The never-smoking patients harbored all the gene fusions in the cohort. The frequencies of genes mutated in 10% or more of never-smokers are visualized in Fig. 1B. Fig. 2 introduces the alteration frequencies in clinically important genes and illustrates the differences of genomic alterations between never-smokers and smokers.

A history of cigarette smoking was associated with more alterations of any type in genes including *TP53, KRAS, SPTA1, LRP1B*, and *STK11.* Never-smokers, in contrast, harbored more alterations in *EGFR, FRS2, MDM2*, and *CDKN2B*. (*P*<0.05, data not shown). Eleven patients with a smoking history had *RUNX1T1* amplification, a finding of unknown clinical significance. Four of our tumors with the *RUNX1T1* amplification were predominantly solid in histology, five were predominantly acinar (with two cribriform tumors), one was micropapillary, and one was lepidic. The impact of smoking on the frequency of selected alterations is presented in Supplementary Table S3.

Significantly, 23 (77%) out of 30 tumors found among never-smokers had an alteration for which an FDA- or EMA-approved NSCLC-indicated targeted treatment is currently available. Additional four never-smokers had an alteration potentially targetable in the near future (Table 2).

### Association of genomic alterations with clinicopathological characteristics and survival

The tumors were histologically classified as described in our earlier manuscript [29]. All tumors with any *EGFR* alterations except one case had predominant histological subtypes associated with favorable survival (lepidic, acinar, papillary). Pathogenic or likely pathogenic *EGFR* mutations occurred in 27.7% of tumors with favorable predominant histologic subtypes, but only in 2.9% of tumors with histologic subtypes associated with poor survival (solid, micropapillary, cribriform, fetal) (*P*=0.002). In contrast, known and likely pathogenic alterations in *KRAS* were associated with predominant histological subtypes conferring poor prognosis (*P*=0.035). The majority (72%, 8/11) of the tumors with *MDM2*-*FRS2* co-amplification were predominantly acinar in histology.

After applying the exclusion criteria, 115 stage I–III patients remained in survival analyses. There was no survival difference between genders or between smokers and never-smokers (*P*>0.05, data not shown). Patients with predominant subtype conferring poor prognosis fared worse in OS (HR 2.345, 95%CI 1.1.335–4.120, *P*=0.003) and DSS analyses (HR 2.930, 95%CI 1.535–5.594, *P*=0.001), as expected.

**Fig. 3:**
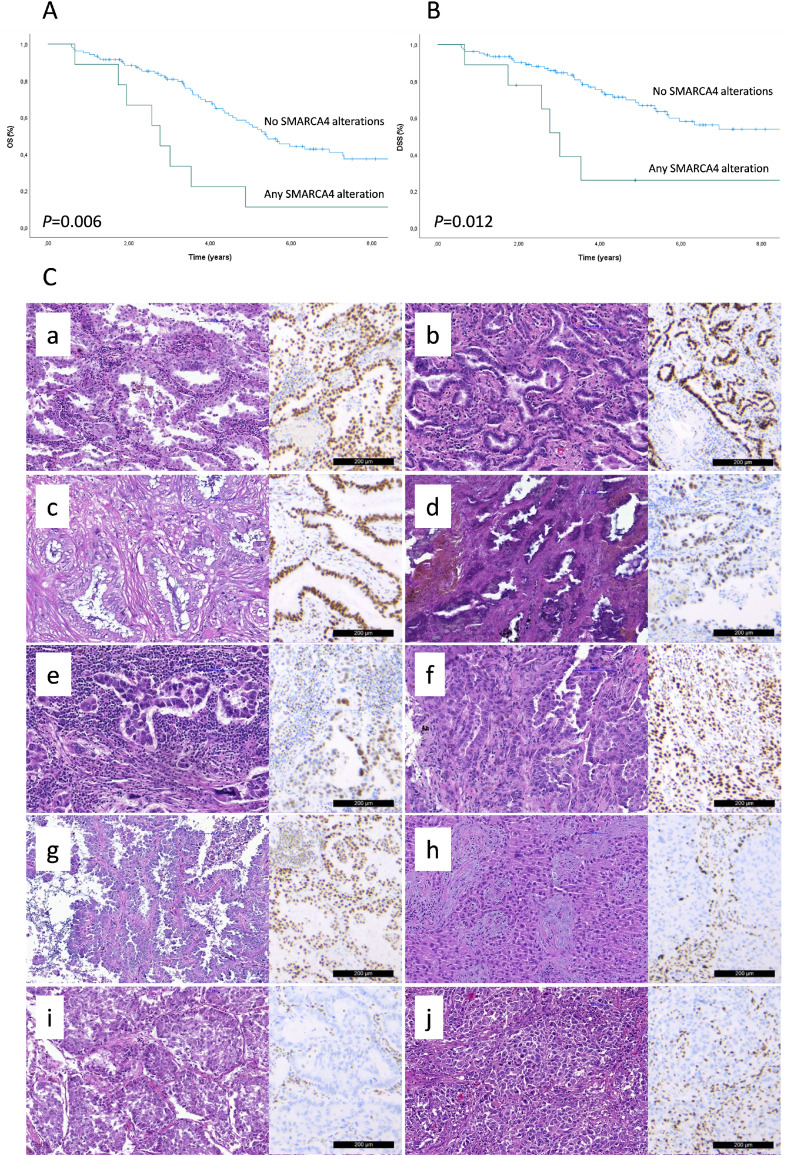
The effect of *SMARCA4* alterations on survival and tumor histology. A) Overall survival (OS) and B) disease specific survival (DSS) demonstrated by Kaplan-Meier analysis. C) Predominant histological subtypes of the *SMARCA4* mutated tumors and the expression of SMARCA4/BRG1 by immunohistochemistry (n=10). Ca–Cf: predominantly acinar tumors, Cg: predominantly papillary tumor, Ch–Cj: predominantly solid tumors. Scale bar 200 µm.

Out of genes relevant to lung adenocarcinoma, any alterations in *SMARCA4* were associated with shortened OS (HR 2.732, 95%CI 1.291-5.782, P=0.009) and DSS (HR 2.901, 95%CI 1.213-6.939, P=0.017) in univariable analysis. The effect of *SMARCA4* alterations on survival is presented in Fig. 3A–B. Alterations of *TP53, KRAS*, or *STK11* were not associated with differences in OS or DSS, either alone or in combination with each other (*P*>0.05). Similarly, the *MDM2-FRS2* co-amplification status was not associated with either OS or DSS (p>0.05).

In multivariable analysis for OS, any alteration in *SMARCA4* (HR 3.522, 95%CI 1.615–7.679, *P*=0.002) was an independent poor prognostic factor together with TMB lower than 14 mutations/MB, and aggressive histologic subgroups. In multivariable analysis for DSS, any alteration in *SMARCA4* (HR 3.911, 95%CI 1.561–9.795, *P*=0.004) was associated with unfavorable outcome along with high stage, TMB lower than 14 mutations/MB, and aggressive histologic subgroups. (Table 3).

**Table 3:** Survival analyses. A. Overall survival and B. disease specific survival analyzed by multivariable Cox analysis. HR=hazard ratio, CI=confidence interval, REF=reference, TMB=tumor mutation burden.

A. Overall survival			
	HR	95%CI	*P-*value
Stage I	REF	REF	REF
Stage II	1.767	0.927–3.366	0.084
Stage III	1.934	1.027–3.644	0.041
Histology: good prognosis	REF	REF	REF
Histology: poor prognosis	2.783	1.485–5.212	0.001
TMB > 14	REF	REF	REF
TMB < 14	2.240	1.210–4.148	0.01
No *SMARCA4* alterations	REF	REF	REF
Any *SMARCA4* alteration	3.522	1.615–7.679	0.002
**B. Disease specific survival**			
	HR	95%CI	*P-*value
Stage I	REF	REF	REF
Stage II	2.375	1.087–5.189	0.03
Stage III	2.882	1.355–6.132	0.006
Histology: good prognosis	REF	REF	REF
Histology: poor prognosis	3.322	1.607–6.866	0.001
TMB > 14	REF	REF	REF
TMB < 14	3.176	1.409–7.158	0.005
No *SMARCA4* alterations	REF	REF	REF
Any *SMARCA4* alteration	3.911	1.561–9.795	0.004

*SMARCA4* alterations were present in ten individual tumors. Seven of these tumors had a differentiated predominant histological subtype (acinar in six and papillary in one, Fig. 3 Ca–Cg), whereas the three remaining tumors were predominantly solid (Fig. 3 Ch–Cj). The tumors with *SMARCA4* alterations were composed of more than one histological subtype, except for one uniformly solid tumor (Fig. 3, patient Ch). Seven tumors harbored *SMARCA4* missense variants, two of which had a concurrent truncating *SMARCA4* mutation. Two tumors had a truncating *SMARCA4* variant without missense variants, and one tumor had a partial *SMARCA4* deletion. The clinicopathological characteristics of the individual tumors with *SMARCA4* alterations are presented in Supplementary Table S4.

All the tumors with *SMARCA4* alterations were further immunohistochemically stained for SMARCA4/BRG1 (Fig. 3 Ca–Cj). The tumors with acinar or papillary predominant histology demonstrated either diffusely positive BRG1 staining (patients Ca-c, Cf–g) or heterogeneous staining including both positive and negative tumor cells (patients Cd–e). The solid predominant tumors (patients Ch–j), all harboring truncating *SMARCA4* variants, had solid (patients Ch–j) and acinar (patient Ci) tumor areas that were BRG1 negative. Adjacent to these negative areas, the tumors exhibited either patchy positive solid areas (patient i) or diffusely positive lepidic growth (patient Cj) suggesting intratumoral heterogeneity in *SMARCA4* mutation status.

Immunohistochemical TTF-1 staining was available from nine *SMARCA4* altered tumors. One tumor with acinar predominant histology and a missense *SMARCA4* variant (patient Cd) and one with solid predominant histology and a combination of truncating and missense *SMARCA4* variants (patient Ci) were TTF-1 negative, while the remaining tumors were TTF-1 positive (Supplementary Table S4).

## Discussion

In estimating the actionability of genome profiling results with the 315 gene panel, we primarily focused on alterations or genomic signatures for which a drug is currently (March 2022) approved for NSCLC by the FDA and/or EMA as they reflect the true actionability in the routine clinical setting. In the overall cohort, the majority (70%) of patients had such an alteration or signature dominated by *EGFR* alterations and TMB ≥ 10. The fraction of patients with actionable alterations remained substantially high (42%) even when excluding TMB. In our cohort, the majority of samples (73%) were from early stage (stages I and II) disease, complicating comparisons to other cohorts with more advanced disease. However, the overall frequencies of actionable alterations were comparable to those reported by Skoulidis and Heymach [30] for metastatic rather than early-stage disease with the exceptions of *ALK* alterations that were close to those reported for early-stage disease, and *EGFR* that falls between those reported for early and metastatic disease. Understanding the mutational landscape in the early-stage setting is becoming increasingly important given that several ongoing trials are investigating targeted drugs in the early-stage setting, and osimertinib has already been approved in the adjuvant setting by the FDA.

In the never-smoker sub-cohort, 77% of patients had a targetable alteration, dominated by alterations in *EGFR*. There were no *KRAS* Gly12Cys mutations among never-smokers, whereas all *ALK, ROS1*, and *RET* fusions were detected exclusively among never-smokers. If the potentially targetable *ERBB2* exon 20 insertions [4,5] are included, the fraction of never-smokers with a targetable alteration in the cohort was 90%. This is in line with a recent study reporting 80% of the never-smokers harboring an actionable alteration [17]. These findings suggest that it is highly likely to find tailored targeted therapy for most, if not all never-smokers with newly diagnosed lung adenocarcinoma.

In survival analyses, we previously identified high TMB (defined in our study as equal to or more than 14 mutations/MB of coding DNA) as a stage- and histology-independent favorable prognostic factor with follow-up data until the end of 2016 [29]. The significance of these parameters in this model persisted with updated survival data extending until the end of 2018. Additionally, any alterations in *SMARCA4*, a gene participating in chromatin remodeling, conferred an independent poor prognostic effect in multivariable analysis. Although the number of patients with *SMARCA4* alterations in our cohort is small, our results support the earlier observations that *SMARCA4* alterations are indicators of poor survival in NSCLC [31], [32], [33], [34].

The *SMARCA4* gene codes SMARCA4/BRG1, an ATP-dependent catalytic subunit of the SWI/SFN chromatin remodeling complex. Most *SMARCA4* alterations in NSCLC are missense variants [33,35]. Truncating mutations, reportedly present in more than one-third of *SMARCA4* mutated tumors, are linked to loss of BRG1 expression indicating BRG1 deficiency, whereas missense mutations mostly leave BRG1 expression intact [36]. Both alteration types are associated with poor clinical outcomes [33], and missense variants seem to affect chromatin remodeling activity [35] in ways other than loss of BRG1 expression.

The majority of the reports evaluating the histopathology of *SMARCA4* altered lung adenocarcinomas have concentrated in tumors with loss of immunohistochemical BRG1 expression [37], [38], [39], [40] and, presumably truncating *SMARCA4* variants. These tumors are poorly differentiated, have a TTF-1 negative status, and behave aggressively. In contrast, the majority of our *SMARCA4* altered tumors exhibited a differentiated predominant subtype, diffuse BRG1 expression, and diffuse positivity for TTF-1. In conclusion, all *SMARCA4* variant types were associated with poor prognosis while tumors with SMARCA4 missense variants were mostly associated with differentiated predominant histological subtypes, retained BRG1 expression, and a positive TTF-1 status.

The *SMARCA4* alteration prevalence in NSCLC is approximately 10% [33,36]. Our patients had a comparable prevalence of 7.4%. In addition to *SMARCA4* alterations providing prognostic information, immunotherapy has yielded some promising results in *SMARCA4* deficient tumors with loss of BRG1 [33,^39^,^41^,42]. In contrast, tumors with *SMARCA4* missense variants did not exhibit a survival benefit on immunotherapy [33]. Furthermore, *SMARCA4* deficient tumors have demonstrated sensitivity to cisplatin-based regimes [31] and, in preclinical studies, to an *ATR* inhibitor [43], a *KDM6* inhibitor [44], and a CDK4/6 inhibitor palbociclib [45]. After more validation, *SMARCA4* may be informative in clinical practice as a prognostic and predictive biomarker. More data are required on whether *SMARCA4* missense variants are clinically actionable.

The Finnish population is genetically unique among Europeans due to historical population bottlenecks, and thus population databases such as The Exome Aggregation Consortium (ExAC) and the Genome Aggregation Database (GNOMad) divide Europeans into Finns or non-Finns. To our knowledge, no comprehensive genomic profiling data is available on Finnish lung adenocarcinoma patients. While most of the genes relevant to lung cancer exhibited similar mutational frequencies compared to non-Finn European populations, the frequency of certain alterations was higher in our cohort than in referenced cohorts. These included *EGFR* (30% vs 7.5–19.0%), *ERBB2* (11.9% vs 1.0–5.0%), *PIK3CA* (10.4% vs 3.8–6.4%), and *BRAF* (8.4% vs 2.9–8.3%) [32,[46], [47], [48]. When comparing the never-smoking sub-cohort with never-smoking non-Finn Europeans, alterations in *TP53* (30% vs 5.8–26.7% [12,^15^,16]) and *ERBB2* (16.7% vs. 3.0-6.7% [14], [15], [16], [17]) were more frequent in our study. An alteration of uncertain significance, *RUNX1T1* amplification, was present in eleven patients, all with a smoking history. One group has previously reported *RUNX1T1* amplification in combined small cell and non-small cell lung cancer [49]. Interestingly, none of our tumors had a neuroendocrine component previously reported for *RUNX1T1* amplified tumors [49]. Further research may elucidate the clinical meaning of the amplification.

Remarkably, only one of our patients had an *ALK* fusion (0.7%), a considerably low number compared to the reported prevalence of 2–7% in other European populations [50]. Furthermore, the pathogenic non-Val600 *BRAF* mutations seem to be more common in Finnish patients than in comparable non-Finn European populations, a finding supported by a previous report [9]. However, as the number of patients in our cohort is small, these findings require further validation with a larger cohort.

## Conclusions

This study broadens the information on genomic alterations in European and Finnish lung adenocarcinoma patients. The most clinically relevant finding was the large number of actionable alterations found among never-smoking patients, supporting using broad genomic profiling, especially if standard tests are negative. *SMARCA4* alterations were stage-, histology- and TMB-independent markers of poor prognosis and were associated with differentiated phenotypes and TTF-1 positivity. *BRAF* non-Val600 mutations and *RUNX1T1* amplifications seem to be unusually prevalent and *ALK* fusions rare in Finnish lung adenocarcinoma patients.

The strengths of the study include the use of the same analysis method on all the tumors and comprehensive follow-up data with up-to-date staging and histological classification. The main limitations are a retrospective cohort and a small number of patients.

## CRediT authorship contribution statement

**Eva-Maria Talvitie:** Conceptualization, Formal analysis, Investigation, Writing – original draft, Visualization. **Lassi Liljeroos:** Conceptualization, Formal analysis, Writing – original draft. **Heikki Vilhonen:** Investigation, Writing – review & editing. **Katri Orte:** Conceptualization, Writing – review & editing. **Ilmo Leivo:** Writing – review & editing. **Markku Kallajoki:** Writing – review & editing. **Pekka Taimen:** Conceptualization, Supervision, Project administration, Writing – review & editing.

## Declaration of Competing Interest

Dr. Talvitie reports personal fees from Roche Oy during the conduct of the study. Dr. Liljeroos is an employee of Roche Oy and has equity interest in F. Hoffmann La Roche AG. Dr. Vilhonen reports grants from Roche Oy during the conduct of the study; personal fees from Roche Oy, personal fees and non-financial support from Takeda, personal fees and non-financial support from Boehringer Ingelheim, outside the submitted work. Dr. Orte reports personal fees from Bayer, non-financial support from Novartis, non-financial support from Astra Zeneca, outside the submitted work. Dr. Kallajoki reports personal fees from Roche Oy, personal fees from Bayer, outside the submitted work. Dr. Taimen reports personal fees from Roche Oy, outside the submitted work.

## Appendix Supplementary materials

Image, application 1

Image, application 2

Image, application 3

Image, application 4
