# Supplementary material for: Comprehensive genomic profiling of Finnish lung adenocarcinoma cohort reveals high clinical actionability and SMARCA4 altered tumors with variable histology and poor prognosis
Source: Neoplasia. 2022 Aug 11;32:100832. doi: 10.1016/j.neo.2022.100832 (PMC9391575; doi:10.1016/j.neo.2022.100832)
Supplement: Supplementary file 1 [file mmc1.docx]

**Supplementary Table 1.** Frequency of individual alterations (n=2682) in 135 lung adenocarcinomas (4–99 alterations per patient).

| Gene | No. of  all individual alterations | No. of known pathogenic alterations | No. of  likely pathogenic alterations | No. of variants of unknown significance | No. of patients with alteration(s) in gene | Percentage of patients with any alteration(s) in gene |
| --- | --- | --- | --- | --- | --- | --- |
| *ABL1* | 7 | 2 | 0 | 5 | 7 | 5.2 |
| *ABL2* | 11 | 0 | 0 | 11 | 10 | 7.4 |
| *ACVR1B* | 1 | 0 | 0 | 1 | 1 | 0.7 |
| *AKT1* | 1 | 1 | 0 | 0 | 1 | 0.7 |
| *AKT2* | 3 | 1 | 0 | 2 | 3 | 2.2 |
| *AKT3* | 3 | 1 | 0 | 2 | 3 | 2.2 |
| *ALK* | 13 | 1 | 0 | 12 | 11 | 8.1 |
| *ALOX12B* | 1 | 0 | 0 | 1 | 1 | 0.7 |
| *APC* | 12 | 2 | 2 | 8 | 11 | 8.1 |
| *APCDD1* | 6 | 0 | 0 | 6 | 6 | 4.4 |
| *AR* | 12 | 0 | 0 | 12 | 10 | 7.4 |
| *ARAF* | 1 | 0 | 0 | 1 | 1 | 0.7 |
| *ARFRP1* | 2 | 0 | 0 | 2 | 2 | 1.5 |
| *ARID1A* | 24 | 2 | 5 | 17 | 19 | 14.1 |
| *ARID1B* | 33 | 0 | 2 | 31 | 23 | 17.0 |
| *ARID2* | 10 | 2 | 2 | 6 | 9 | 6.7 |
| *ASXL1* | 4 | 1 | 0 | 3 | 4 | 3.0 |
| *ATM* | 24 | 5 | 6 | 13 | 19 | 14.1 |
| *ATR* | 10 | 0 | 0 | 10 | 9 | 6.7 |
| *ATRX* | 12 | 0 | 0 | 12 | 10 | 7.4 |
| *AURKA* | 2 | 1 | 0 | 1 | 2 | 1.5 |
| *AXIN1* | 4 | 0 | 0 | 4 | 4 | 3.0 |
| *AXL* | 8 | 0 | 0 | 8 | 8 | 5.9 |
| *BACH1* | 5 | 0 | 0 | 5 | 5 | 3.7 |
| *BAP1* | 2 | 0 | 1 | 1 | 2 | 1.5 |
| *BARD1* | 9 | 0 | 1 | 8 | 9 | 6.7 |
| *BCL2L1* | 1 | 0 | 0 | 1 | 1 | 0.7 |
| *BCL6* | 1 | 0 | 0 | 1 | 1 | 0.7 |
| *BCOR* | 7 | 0 | 1 | 6 | 7 | 5.2 |
| *BCORL1* | 15 | 2 | 0 | 13 | 12 | 8.9 |
| *BCR* | 1 | 0 | 0 | 1 | 1 | 0.7 |
| *BLM* | 5 | 0 | 0 | 5 | 5 | 3.7 |
| *BRAF* | 13 | 9 | 1 | 3 | 12 | 8.9 |
| *BRCA1* | 4 | 0 | 1 | 3 | 4 | 3.0 |
| *BRCA2* | 8 | 2 | 1 | 5 | 8 | 5.9 |
| *BRD4* | 7 | 0 | 1 | 6 | 7 | 5.2 |
| *BRIP1* | 3 | 0 | 1 | 2 | 3 | 2.2 |
| *BTG1* | 1 | 0 | 0 | 1 | 1 | 0.7 |
| *BTK* | 5 | 0 | 0 | 5 | 5 | 3.7 |
| *CARD11* | 6 | 0 | 0 | 6 | 5 | 3.7 |
| *CASP8* | 2 | 0 | 0 | 2 | 2 | 1.5 |
| *CBFB* | 1 | 0 | 0 | 1 | 1 | 0.7 |
| *CBL* | 3 | 0 | 1 | 2 | 3 | 2.2 |
| *CCND1* | 3 | 2 | 0 | 1 | 3 | 2.2 |
| *CCND2* | 3 | 1 | 0 | 2 | 3 | 2.2 |
| *CCND3* | 3 | 3 | 0 | 0 | 3 | 2.2 |
| *CCNE1* | 4 | 3 | 0 | 1 | 4 | 3.0 |
| *CD274* | 4 | 2 | 0 | 2 | 4 | 3.0 |
| *CD79A* | 1 | 0 | 0 | 1 | 1 | 0.7 |
| *CD79B* | 2 | 0 | 0 | 2 | 2 | 1.5 |
| *CDC73* | 4 | 0 | 0 | 4 | 4 | 3.0 |
| *CDH1* | 3 | 1 | 0 | 2 | 3 | 2.2 |
| *CDH2* | 13 | 0 | 0 | 13 | 10 | 7.4 |
| *CDH20* | 9 | 1 | 0 | 8 | 8 | 5.9 |
| *CDH5* | 8 | 0 | 0 | 8 | 7 | 5.2 |
| *CDK12* | 8 | 0 | 1 | 7 | 8 | 5.9 |
| *CDK4* | 8 | 7 | 0 | 1 | 8 | 5.9 |
| *CDK6* | 1 | 0 | 0 | 1 | 1 | 0.7 |
| *CDK8* | 1 | 0 | 0 | 1 | 1 | 0.7 |
| *CDKN1A* | 2 | 0 | 0 | 2 | 2 | 1.5 |
| *CDKN1B* | 3 | 0 | 1 | 2 | 3 | 2.2 |
| *CDKN2A* | 23 | 17 | 1 | 5 | 23 | 17.0 |
| *CDKN2B* | 16 | 14 | 0 | 2 | 16 | 11.9 |
| *CEBPA* | 4 | 0 | 0 | 4 | 4 | 3.0 |
| *CHD2* | 3 | 0 | 0 | 3 | 3 | 2.2 |
| *CHD4* | 5 | 0 | 1 | 4 | 4 | 3.0 |
| *CHEK1* | 3 | 0 | 0 | 3 | 3 | 2.2 |
| *CHEK2* | 5 | 3 | 0 | 2 | 5 | 3.7 |
| *CIC* | 2 | 0 | 1 | 1 | 2 | 1.5 |
| *CRBN* | 1 | 0 | 0 | 1 | 1 | 0.7 |
| *CREBBP* | 10 | 0 | 0 | 10 | 10 | 7.4 |
| *CRKL* | 3 | 1 | 0 | 2 | 2 | 1.5 |
| *CRLF2* | 3 | 0 | 0 | 3 | 3 | 2.2 |
| *CSF1R* | 7 | 0 | 0 | 7 | 7 | 5.2 |
| *CTCF* | 4 | 0 | 0 | 4 | 4 | 3.0 |
| *CTNNA1* | 6 | 0 | 0 | 6 | 6 | 4.4 |
| *CTNNB1* | 6 | 5 | 0 | 1 | 6 | 4.4 |
| *CUL3* | 4 | 0 | 0 | 4 | 4 | 3.0 |
| *CUL4B* | 6 | 1 | 0 | 5 | 5 | 3.7 |
| *CYLD* | 2 | 0 | 0 | 2 | 2 | 1.5 |
| *CYP17A1* | 1 | 0 | 0 | 1 | 1 | 0.7 |
| *DAXX* | 2 | 0 | 1 | 1 | 2 | 1.5 |
| *DDR1* | 8 | 0 | 0 | 8 | 8 | 5.9 |
| *DDR2* | 6 | 0 | 0 | 6 | 6 | 4.4 |
| *DICER1* | 7 | 0 | 1 | 6 | 7 | 5.2 |
| *DIS3* | 10 | 0 | 0 | 10 | 9 | 6.7 |
| *DNMT3A* | 5 | 2 | 1 | 2 | 5 | 3.7 |
| *DOT1L* | 3 | 0 | 0 | 3 | 3 | 2.2 |
| *EGFR* | 41 | 35 | 1 | 5 | 32 | 23.7 |
| *EMSY* | 3 | 1 | 0 | 2 | 3 | 2.2 |
| *EP300* | 18 | 0 | 1 | 17 | 18 | 13.3 |
| *EPHA3* | 20 | 0 | 0 | 20 | 17 | 12.6 |
| *EPHA5* | 17 | 2 | 0 | 15 | 13 | 9.6 |
| *EPHA6* | 11 | 0 | 0 | 11 | 8 | 5.9 |
| *EPHA7* | 4 | 0 | 0 | 4 | 3 | 2.2 |
| *EPHB1* | 14 | 0 | 0 | 14 | 13 | 9.6 |
| *EPHB4* | 7 | 0 | 0 | 7 | 7 | 5.2 |
| *EPHB6* | 11 | 0 | 0 | 11 | 10 | 7.4 |
| *ERBB2* | 16 | 8 | 0 | 8 | 14 | 10.4 |
| *ERBB3* | 7 | 2 | 0 | 5 | 6 | 4.4 |
| *ERBB4* | 10 | 0 | 0 | 10 | 10 | 7.4 |
| *ERCC4* | 4 | 0 | 0 | 4 | 4 | 3.0 |
| *ERG* | 1 | 0 | 0 | 1 | 1 | 0.7 |
| *ERRFI1* | 1 | 0 | 1 | 0 | 1 | 0.7 |
| *ESR1* | 11 | 1 | 0 | 10 | 10 | 7.4 |
| *FAM123B* | 6 | 0 | 0 | 6 | 5 | 3.7 |
| *FAM175A* | 1 | 0 | 0 | 1 | 1 | 0.7 |
| *FAM46C* | 1 | 0 | 0 | 1 | 1 | 0.7 |
| *FANCA* | 7 | 0 | 1 | 6 | 7 | 5.2 |
| *FANCC* | 1 | 0 | 0 | 1 | 1 | 0.7 |
| *FANCD2* | 2 | 0 | 0 | 2 | 2 | 1.5 |
| *FANCE* | 5 | 0 | 0 | 5 | 5 | 3.7 |
| *FANCF* | 3 | 0 | 1 | 2 | 3 | 2.2 |
| *FANCG* | 7 | 0 | 2 | 5 | 7 | 5.2 |
| *FANCI* | 1 | 0 | 0 | 1 | 1 | 0.7 |
| *FANCL* | 3 | 0 | 0 | 3 | 3 | 2.2 |
| *FANCM* | 11 | 0 | 0 | 11 | 10 | 7.4 |
| *FAS* | 4 | 0 | 1 | 3 | 4 | 3.0 |
| *FAT1* | 21 | 0 | 1 | 20 | 19 | 14.1 |
| *FAT3* | 43 | 0 | 0 | 43 | 26 | 19.3 |
| *FBXW7* | 2 | 0 | 0 | 2 | 2 | 1.5 |
| *FGF10* | 8 | 4 | 0 | 4 | 8 | 5.9 |
| *FGF12* | 1 | 0 | 0 | 1 | 1 | 0.7 |
| *FGF14* | 3 | 0 | 0 | 3 | 3 | 2.2 |
| *FGF19* | 4 | 1 | 0 | 3 | 4 | 3.0 |
| *FGF23* | 4 | 1 | 0 | 3 | 3 | 2.2 |
| *FGF3* | 1 | 1 | 0 | 0 | 1 | 0.7 |
| *FGF4* | 3 | 1 | 0 | 2 | 3 | 2.2 |
| *FGF6* | 4 | 1 | 0 | 3 | 4 | 3.0 |
| *FGFR1* | 6 | 1 | 0 | 5 | 5 | 3.7 |
| *FGFR3* | 4 | 0 | 0 | 4 | 4 | 3.0 |
| *FGFR4* | 3 | 0 | 0 | 3 | 3 | 2.2 |
| *FH* | 3 | 0 | 0 | 3 | 3 | 2.2 |
| *FLCN* | 6 | 0 | 3 | 3 | 6 | 4.4 |
| *FLT1* | 24 | 0 | 0 | 24 | 23 | 17.0 |
| *FLT3* | 4 | 0 | 0 | 4 | 4 | 3.0 |
| *FLT4* | 18 | 0 | 0 | 18 | 15 | 11.1 |
| *FRS2* | 11 | 10 | 0 | 1 | 11 | 8.1 |
| *FUBP1* | 3 | 0 | 0 | 3 | 3 | 2.2 |
| *GABRA6* | 8 | 1 | 0 | 7 | 8 | 5.9 |
| *GALNT12* | 2 | 0 | 0 | 2 | 2 | 1.5 |
| *GATA1* | 4 | 0 | 0 | 4 | 4 | 3.0 |
| *GATA2* | 5 | 0 | 1 | 4 | 5 | 3.7 |
| *GATA3* | 3 | 0 | 0 | 3 | 2 | 1.5 |
| *GATA4* | 1 | 0 | 0 | 1 | 1 | 0.7 |
| *GATA6* | 5 | 0 | 0 | 5 | 5 | 3.7 |
| *GEN1* | 1 | 0 | 0 | 1 | 1 | 0.7 |
| *GLI1* | 10 | 2 | 0 | 8 | 10 | 7.4 |
| *GNA11* | 2 | 0 | 0 | 2 | 2 | 1.5 |
| *GNA13* | 2 | 0 | 0 | 2 | 2 | 1.5 |
| *GNAQ* | 1 | 0 | 0 | 1 | 1 | 0.7 |
| *GNAS* | 9 | 2 | 0 | 7 | 8 | 5.9 |
| *GPR124* | 19 | 0 | 0 | 19 | 19 | 14.1 |
| *GREM1* | 2 | 0 | 0 | 2 | 2 | 1.5 |
| *GRIN2A* | 15 | 1 | 0 | 14 | 14 | 10.4 |
| *GRM3* | 6 | 0 | 0 | 6 | 5 | 3.7 |
| *GSK3B* | 1 | 0 | 0 | 1 | 1 | 0.7 |
| *H3F3A* | 1 | 0 | 0 | 1 | 1 | 0.7 |
| *HGF* | 15 | 2 | 0 | 13 | 14 | 10.4 |
| *HNF1A* | 1 | 0 | 0 | 1 | 1 | 0.7 |
| *HOXB13* | 4 | 0 | 0 | 4 | 4 | 3.0 |
| *HSD3B1* | 2 | 0 | 0 | 2 | 2 | 1.5 |
| *HSP90AA1* | 13 | 0 | 0 | 13 | 12 | 8.9 |
| *IDH1* | 5 | 2 | 0 | 3 | 5 | 3.7 |
| *IGF1* | 1 | 0 | 0 | 1 | 1 | 0.7 |
| *IGF1R* | 9 | 0 | 0 | 9 | 9 | 6.7 |
| *IGF2* | 1 | 0 | 0 | 1 | 1 | 0.7 |
| *IGF2R* | 2 | 1 | 0 | 1 | 2 | 1.5 |
| *IKBKE* | 6 | 0 | 0 | 6 | 6 | 4.4 |
| *IKZF1* | 2 | 0 | 0 | 2 | 2 | 1.5 |
| *IL7R* | 23 | 1 | 0 | 22 | 21 | 15.6 |
| *INHBA* | 6 | 0 | 0 | 6 | 5 | 3.7 |
| *INPP4B* | 11 | 0 | 2 | 9 | 10 | 7.4 |
| *INSR* | 4 | 0 | 0 | 4 | 4 | 3.0 |
| *IRF2* | 4 | 0 | 2 | 2 | 4 | 3.0 |
| *IRF4* | 5 | 0 | 0 | 5 | 5 | 3.7 |
| *IRS2* | 14 | 0 | 0 | 14 | 12 | 8.9 |
| *JAK1* | 1 | 0 | 0 | 1 | 1 | 0.7 |
| *JAK2* | 9 | 2 | 0 | 7 | 9 | 6.7 |
| *JAK3* | 6 | 0 | 0 | 6 | 6 | 4.4 |
| *JUN* | 1 | 0 | 0 | 1 | 1 | 0.7 |
| *KDM5A* | 9 | 2 | 0 | 7 | 7 | 5.2 |
| *KDM5C* | 9 | 0 | 1 | 8 | 7 | 5.2 |
| *KDM6A* | 3 | 0 | 2 | 1 | 3 | 2.2 |
| *KDR* | 15 | 0 | 0 | 15 | 14 | 10.4 |
| *KEAP1* | 19 | 1 | 4 | 14 | 19 | 14.1 |
| *KEL* | 9 | 0 | 1 | 8 | 7 | 5.2 |
| *KIT* | 3 | 0 | 0 | 3 | 3 | 2.2 |
| *KLHL6* | 1 | 0 | 0 | 1 | 1 | 0.7 |
| *KRAS* | 57 | 53 | 0 | 4 | 51 | 37.8 |
| *LMO1* | 3 | 0 | 0 | 3 | 3 | 2.2 |
| *LRP1B* | 65 | 3 | 12 | 50 | 38 | 28.1 |
| *LRP6* | 5 | 1 | 0 | 4 | 5 | 3.7 |
| *LTK* | 5 | 0 | 0 | 5 | 4 | 3.0 |
| *LYN* | 6 | 4 | 0 | 2 | 6 | 5.2 |
| *LZTR1* | 12 | 0 | 2 | 10 | 12 | 8.9 |
| *MAGI2* | 7 | 0 | 1 | 6 | 7 | 5.2 |
| *MAP2K1* | 1 | 0 | 0 | 1 | 1 | 0.7 |
| *MAP2K2* | 1 | 0 | 0 | 1 | 1 | 0.7 |
| *MAP2K4* | 3 | 0 | 2 | 1 | 3 | 2.2 |
| *MAP3K1* | 10 | 0 | 0 | 10 | 10 | 7.4 |
| *MAP3K13* | 5 | 0 | 0 | 5 | 4 | 3.0 |
| *MCL1* | 1 | 1 | 0 | 0 | 1 | 0.7 |
| *MDM2* | 10 | 10 | 0 | 0 | 10 | 7.4 |
| *MDM4* | 1 | 1 | 0 | 0 | 1 | 0.7 |
| *MED12* | 17 | 0 | 0 | 17 | 17 | 12.6 |
| *MEF2B* | 1 | 0 | 0 | 1 | 1 | 0.7 |
| *MEN1* | 1 | 0 | 0 | 1 | 1 | 0.7 |
| *MERTK* | 2 | 0 | 0 | 2 | 2 | 1.5 |
| *MET* | 14 | 5 | 4 | 5 | 13 | 9.6 |
| *MITF* | 5 | 0 | 0 | 5 | 5 | 3.7 |
| *MKNK1* | 7 | 0 | 0 | 7 | 7 | 5.2 |
| *MKNK2* | 4 | 0 | 0 | 4 | 4 | 3.0 |
| *MLH1* | 4 | 0 | 1 | 3 | 4 | 3.0 |
| *MLL* | 7 | 0 | 0 | 7 | 6 | 4.4 |
| *MLL2* | 21 | 0 | 6 | 15 | 20 | 14.8 |
| *MLL3* | 24 | 1 | 6 | 17 | 22 | 16.3 |
| *MPL* | 7 | 0 | 0 | 7 | 7 | 5.2 |
| *MRE11A* | 4 | 0 | 1 | 3 | 4 | 3.0 |
| *MSH2* | 4 | 0 | 2 | 2 | 4 | 3.0 |
| *MSH6* | 3 | 1 | 2 | 0 | 3 | 2.2 |
| *MST1R* | 2 | 0 | 0 | 2 | 2 | 1.5 |
| *MTOR* | 6 | 0 | 0 | 6 | 5 | 3.7 |
| *MUTYH* | 4 | 2 | 0 | 2 | 4 | 3.0 |
| *MYC* | 10 | 9 | 0 | 1 | 10 | 7.4 |
| *MYCL1* | 5 | 1 | 0 | 4 | 3 | 2.2 |
| *MYCN* | 2 | 0 | 0 | 2 | 2 | 1.5 |
| *MYD88* | 2 | 0 | 0 | 2 | 2 | 1.5 |
| *MYST3* | 23 | 2 | 0 | 21 | 23 | 17.0 |
| *NBN* | 13 | 0 | 0 | 13 | 13 | 9.6 |
| *NCOR1* | 5 | 0 | 0 | 5 | 5 | 3.7 |
| *NF1* | 20 | 1 | 10 | 9 | 14 | 10.4 |
| *NF2* | 4 | 1 | 0 | 3 | 4 | 3.0 |
| *NFE2L2* | 8 | 2 | 0 | 6 | 8 | 5.9 |
| *NFKBIA* | 3 | 1 | 2 | 0 | 3 | 2.2 |
| *NKX2-1* | 6 | 3 | 0 | 3 | 6 | 4.4 |
| *NOTCH1* | 15 | 2 | 1 | 12 | 13 | 9.6 |
| *NOTCH2* | 18 | 2 | 2 | 14 | 15 | 11.1 |
| *NOTCH3* | 5 | 0 | 1 | 4 | 5 | 3.7 |
| *NOTCH4* | 15 | 0 | 2 | 13 | 12 | 8.9 |
| *NPM1* | 3 | 0 | 0 | 3 | 3 | 2.2 |
| *NRAS* | 4 | 2 | 0 | 2 | 4 | 3.0 |
| *NSD1* | 7 | 0 | 0 | 7 | 7 | 5.2 |
| *NTRK1* | 9 | 0 | 0 | 9 | 9 | 6.7 |
| *NTRK2* | 1 | 0 | 0 | 1 | 1 | 0.7 |
| *NTRK3* | 15 | 0 | 0 | 15 | 14 | 10.4 |
| *NUP93* | 3 | 0 | 0 | 3 | 3 | 2.2 |
| *PAK3* | 3 | 0 | 0 | 3 | 3 | 2.2 |
| *PAK7* | 11 | 0 | 0 | 11 | 7 | 5.2 |
| *PALB2* | 5 | 0 | 0 | 5 | 5 | 3.7 |
| *PARK2* | 4 | 0 | 0 | 4 | 4 | 3.0 |
| *PARP1* | 6 | 0 | 0 | 6 | 6 | 4.4 |
| *PARP2* | 3 | 0 | 0 | 3 | 3 | 2.2 |
| *PARP3* | 2 | 0 | 0 | 2 | 2 | 1.5 |
| *PARP4* | 4 | 1 | 0 | 3 | 4 | 3.0 |
| *PAX5* | 2 | 0 | 1 | 1 | 2 | 1.5 |
| *PBRM1* | 3 | 0 | 1 | 2 | 3 | 2.2 |
| *PDCD1LG2* | 2 | 2 | 0 | 0 | 2 | 1.5 |
| *PDGFRA* | 14 | 0 | 0 | 14 | 11 | 8.1 |
| *PDGFRB* | 10 | 0 | 0 | 10 | 10 | 7.4 |
| *PDK1* | 1 | 0 | 0 | 1 | 1 | 0.7 |
| *PHLPP2* | 4 | 0 | 0 | 4 | 4 | 3.0 |
| *PIK3C2B* | 12 | 1 | 0 | 11 | 11 | 8.1 |
| *PIK3C2G* | 21 | 1 | 0 | 20 | 19 | 14.1 |
| *PIK3C3* | 7 | 0 | 0 | 7 | 7 | 5.2 |
| *PIK3CA* | 14 | 12 | 0 | 2 | 11 | 8.1 |
| *PIK3CB* | 5 | 2 | 0 | 3 | 5 | 3.7 |
| *PIK3CG* | 9 | 0 | 0 | 9 | 9 | 6.7 |
| *PIK3R1* | 1 | 0 | 0 | 1 | 1 | 0.7 |
| *PIK3R2* | 2 | 1 | 0 | 1 | 2 | 1.5 |
| *PLCG2* | 2 | 0 | 0 | 2 | 2 | 1.5 |
| *PMS2* | 6 | 1 | 0 | 5 | 6 | 4.4 |
| *PNRC1* | 3 | 0 | 0 | 3 | 3 | 2.2 |
| *POLD1* | 12 | 0 | 0 | 12 | 12 | 8.9 |
| *POLE* | 13 | 1 | 0 | 12 | 11 | 8.1 |
| *PPARG* | 2 | 0 | 0 | 2 | 2 | 1.5 |
| *PPP2R1A* | 3 | 0 | 0 | 3 | 3 | 2.2 |
| *PRDM1* | 2 | 0 | 0 | 2 | 2 | 1.5 |
| *PREX2* | 27 | 2 | 0 | 25 | 24 | 17.8 |
| *PRKAR1A* | 1 | 0 | 0 | 1 | 1 | 0.7 |
| *PRKCI* | 2 | 0 | 0 | 2 | 2 | 1.5 |
| *PRKDC* | 43 | 0 | 0 | 43 | 38 | 28.1 |
| *PRSS1* | 12 | 0 | 0 | 12 | 11 | 8.1 |
| *PTCH1* | 5 | 0 | 2 | 3 | 5 | 3.7 |
| *PTCH2* | 4 | 0 | 0 | 4 | 4 | 3.0 |
| *PTEN* | 7 | 2 | 1 | 4 | 6 | 4.4 |
| *PTPN11* | 3 | 1 | 0 | 2 | 3 | 2.2 |
| *PTPRD* | 15 | 0 | 5 | 10 | 14 | 10.4 |
| *QKI* | 1 | 0 | 0 | 1 | 1 | 0.7 |
| *RAC1* | 1 | 0 | 0 | 1 | 1 | 0.7 |
| *RAD50* | 6 | 0 | 2 | 4 | 6 | 4.4 |
| *RAD51* | 2 | 0 | 0 | 2 | 2 | 1.5 |
| *RAD51B* | 6 | 0 | 0 | 6 | 6 | 4.4 |
| *RAD51D* | 1 | 0 | 0 | 1 | 1 | 0.7 |
| *RAD52* | 3 | 0 | 0 | 3 | 3 | 2.2 |
| *RAD54L* | 3 | 2 | 0 | 1 | 3 | 2.2 |
| *RAF1* | 1 | 0 | 0 | 1 | 1 | 0.7 |
| *RANBP2* | 8 | 0 | 0 | 8 | 8 | 5.9 |
| *RARA* | 4 | 1 | 0 | 3 | 4 | 3.0 |
| *RB1* | 8 | 1 | 4 | 3 | 8 | 5.9 |
| *RBM10* | 16 | 1 | 13 | 2 | 16 | 11.9 |
| *REL* | 2 | 0 | 0 | 2 | 2 | 1.5 |
| *RET* | 10 | 3 | 1 | 6 | 10 | 7.4 |
| *RICTOR* | 9 | 7 | 0 | 2 | 9 | 6.7 |
| *RNF43* | 10 | 0 | 2 | 8 | 10 | 7.4 |
| *ROS1* | 7 | 1 | 0 | 6 | 7 | 5.2 |
| *RPA1* | 6 | 0 | 0 | 6 | 6 | 4.4 |
| *RPTOR* | 2 | 0 | 0 | 2 | 2 | 1.5 |
| *RUNX1* | 3 | 0 | 1 | 2 | 3 | 2.2 |
| *RUNX1T1* | 21 | 0 | 1 | 20 | 21 | 15.6 |
| *SDHA* | 19 | 1 | 0 | 18 | 18 | 13.3 |
| *SDHC* | 6 | 0 | 0 | 6 | 6 | 4.4 |
| *SETD2* | 9 | 0 | 5 | 4 | 9 | 6.7 |
| *SF3B1* | 5 | 2 | 0 | 3 | 5 | 3.7 |
| *SH2B3* | 5 | 0 | 0 | 5 | 5 | 3.7 |
| *SLIT2* | 15 | 1 | 2 | 12 | 14 | 10.4 |
| *SMAD3* | 3 | 0 | 0 | 3 | 3 | 2.2 |
| *SMAD4* | 6 | 3 | 1 | 2 | 6 | 4.4 |
| *SMARCA4* | 12 | 2 | 7 | 3 | 10 | 7.4 |
| *SMARCB1* | 1 | 0 | 0 | 1 | 1 | 0.7 |
| *SMARCD1* | 2 | 0 | 0 | 2 | 2 | 1.5 |
| *SMO* | 6 | 1 | 0 | 5 | 6 | 4.4 |
| *SNCAIP* | 2 | 0 | 0 | 2 | 2 | 1.5 |
| *SOCS1* | 1 | 0 | 0 | 1 | 1 | 0.7 |
| *SOX10* | 2 | 0 | 0 | 2 | 2 | 1.5 |
| *SOX2* | 2 | 0 | 0 | 2 | 2 | 1.5 |
| *SOX9* | 7 | 0 | 2 | 5 | 7 | 5.2 |
| *SPEN* | 18 | 1 | 0 | 17 | 17 | 12.6 |
| *SPOP* | 4 | 0 | 0 | 4 | 4 | 3.0 |
| *SPTA1* | 48 | 1 | 7 | 40 | 38 | 28.1 |
| *SRC* | 2 | 0 | 0 | 2 | 2 | 1.5 |
| *STAG2* | 1 | 0 | 0 | 1 | 1 | 0.7 |
| *STAT3* | 3 | 1 | 0 | 2 | 2 | 1.5 |
| *STAT4* | 5 | 0 | 1 | 4 | 5 | 3.7 |
| *STK11* | 30 | 9 | 14 | 7 | 27 | 20.0 |
| *SYK* | 7 | 0 | 0 | 7 | 5 | 3.7 |
| *TAF1* | 10 | 0 | 1 | 9 | 9 | 6.7 |
| *TBX3* | 9 | 0 | 0 | 9 | 8 | 5.9 |
| *TEK* | 5 | 0 | 0 | 5 | 5 | 3.7 |
| *TET2* | 6 | 1 | 0 | 5 | 6 | 4.4 |
| *TGFBR2* | 2 | 0 | 0 | 2 | 2 | 1.5 |
| *TNF* | 3 | 0 | 0 | 3 | 3 | 2.2 |
| *TNFAIP3* | 2 | 0 | 1 | 1 | 2 | 1.5 |
| *TNFRSF14* | 2 | 0 | 0 | 2 | 2 | 1.5 |
| *TNKS* | 4 | 0 | 0 | 4 | 4 | 3.0 |
| *TNKS2* | 4 | 0 | 0 | 4 | 4 | 3.0 |
| *TOP1* | 2 | 0 | 0 | 2 | 2 | 1.5 |
| *TOP2A* | 5 | 1 | 0 | 4 | 4 | 3.0 |
| *TP53* | 84 | 59 | 23 | 2 | 74 | 54.8 |
| *TP53BP1* | 5 | 1 | 0 | 4 | 5 | 3.7 |
| *TRRAP* | 16 | 1 | 0 | 15 | 16 | 11.9 |
| *TSC1* | 11 | 0 | 1 | 10 | 11 | 8.1 |
| *TSC2* | 10 | 0 | 1 | 9 | 9 | 6.7 |
| *TSHR* | 8 | 0 | 0 | 8 | 8 | 5.9 |
| *TYRO3* | 4 | 0 | 0 | 4 | 4 | 3.0 |
| *U2AF1* | 2 | 2 | 0 | 0 | 2 | 1.5 |
| *VEGFA* | 5 | 3 | 0 | 2 | 5 | 3.7 |
| *VHL* | 2 | 0 | 0 | 2 | 2 | 1.5 |
| *WISP3* | 1 | 0 | 0 | 1 | 1 | 0.7 |
| *WT1* | 4 | 0 | 1 | 3 | 4 | 3.0 |
| *XPO1* | 1 | 0 | 0 | 1 | 1 | 0.7 |
| *ZBTB2* | 1 | 0 | 0 | 1 | 1 | 0.7 |
| *ZNF217* | 7 | 2 | 0 | 5 | 6 | 4.4 |
| *ZNF703* | 5 | 0 | 0 | 5 | 5 | 3.7 |
| *ZNRF3* | 4 | 0 | 0 | 4 | 4 | 3.0 |
|  |  |  |  |  |  |  |
