# Supplementary material for: Comprehensive genomic profiling of Finnish lung adenocarcinoma cohort reveals high clinical actionability and SMARCA4 altered tumors with variable histology and poor prognosis
Source: Neoplasia. 2022 Aug 11;32:100832. doi: 10.1016/j.neo.2022.100832 (PMC9391575; doi:10.1016/j.neo.2022.100832)
Supplement: Supplementary file 2 [file mmc2.docx]

**Supplementary Table 2.** All genomic alterations in selected genes relevant to lung adenocarcinoma.

| ***EGFR*** | |  |  | | |  | | | |  | | |  | |  | | | |  | | |  | | |  |
| --- | --- | --- | --- | --- | --- | --- | --- | --- | --- | --- | --- | --- | --- | --- | --- | --- | --- | --- | --- | --- | --- | --- | --- | --- | --- |
| **Alteration** | | **Protein change** | | | **cDNA sequence change** | | | | **Known pathogenic** | | | **Likely pathogenic** | | | | **VUS** | | **Total no. of alterations** | | | **No. of alterations in never-smokers** | | | **% of alterations**  **in never-smokers** | |
| Exon 19 deletions | | E746_A750del | | | 2235_2249delGGAATTAAGAGAAGC, 2236_2250delGAATTAAGAGAAGCA | | | | 9 | | | 0 | | | | 0 | | 9 | | | 6 | | | 6 | |
|  |  | L747_P753>Q | | | 2239_2258TTAAGAGAAGCAACATCTCC>CA | | | | 1 | | | 0 | | | | 0 | | 1 | | | 1 | | | 1 | |
|  |  | L747_S752del | | | 2239_2256delTTAAGAGAAGCAACATCT | | | | 1 | | | 0 | | | | 0 | | 1 | | | 0 | | | 0 | |
|  |  | L747_P753>S | | | 2240_2257delTAAGAGAAGCAACATCTC | | | | 2 | | | 0 | | | | 0 | | 2 | | | 0 | | | 0 | |
|  | |  | | |  | | | |  | | |  | | | |  | |  | | |  | | |  | |
| Exon 21 mutation | | L858R | | | 2573T>G | | | | 8 | | | 0 | | | | 0 | | 8 | | | 5 | | | 5 | |
|  | |  | | |  | | | |  | | |  | | | |  | |  | | |  | | |  | |
| Rearrangements | | Amplification | | |  | | | | 4 | | | 0 | | | | 0 | | 4 | | | 2 | | | 2 | |
|  | |  | | |  | | | |  | | |  | | | |  | |  | | |  | | |  | |
| Exon 20 insertions | | H773_V774insNPH | | | 2319_2320insAACCCCCAC | | | | 1 | | | 0 | | | | 0 | | 1 | | | 1 | | | 1 | |
|  |  | V769_D770insASV | | | 2308_2309insCCAGCGTGG | | | | 1 | | | 0 | | | | 0 | | 1 | | | 0 | | | 0 | |
|  |  | N771_P772insG | | | 2313_2314insGGC | | | | 0 | | | 1 | | | | 0 | | 1 | | | 1 | | | 1 | |
|  |  | V774_C775insHV | | | 2322_2323insCACGTG | | | | 1 | | | 0 | | | | 0 | | 1 | | | 1 | | | 1 | |
|  | |  | | |  | | | |  | | |  | | | |  | |  | | |  | | |  | |
| Others | | G719A | | | 2156G>C | | | | 2 | | | 0 | | | | 0 | | 2 | | | 0 | | | 0 | |
|  |  | G719C | | | 2155G>T | | | | 1 | | | 0 | | | | 0 | | 1 | | | 0 | | | 0 | |
|  |  | S768I | | | 2303G>T | | | | 2 | | | 0 | | | | 0 | | 2 | | | 0 | | | 0 | |
|  |  | E709K | | | 2125G>A | | | | 1 | | | 0 | | | | 0 | | 1 | | | 0 | | | 0 | |
|  |  | S811F | | | 2432C>T | | | | 1 | | | 0 | | | | 0 | | 1 | | | 0 | | | 0 | |
|  | |  | | |  | | | |  | | |  | | | |  | |  | | |  | | |  | |
| VUS | | Rearrangements | | |  | | | | 0 | | | 0 | | | | 2 | | 2 | | | 1 | | | 1 | |
|  |  | N196I | | | 587A>T | | | | 0 | | | 0 | | | | 1 | | 1 | | | 0 | | | 0 | |
|  |  | R252H | | | 755G>A | | | | 0 | | | 0 | | | | 1 | | 1 | | | 0 | | | 0 | |
|  |  | R675L | | | 2024_2025GG>TT | | | | 0 | | | 0 | | | | 1 | | 1 | | | 0 | | | 0 | |
|  | |  | | |  | | | |  | | |  | | | |  | |  | | |  | | |  | |
|  | |  | | |  | | | |  | | |  | | | |  | |  | | |  | | |  | |
|  | |  | | |  | | | |  | | |  | | | |  | |  | | |  | | |  | |
| ***KRAS*** | |  | | |  | | | |  | | |  | | | |  | |  | | |  | | |  | |
| **Alteration** | | **Protein change** | | | **Coding DNA sequence change** | | | | **Known pathogenic** | | | **Likely pathogenic** | | | | **VUS** | | **Total n:o of alterations** | | | **No. of alterations in never smokers** | | | **% of alterations**  **in never-smokers** | |
| Codon 12 | | G12C | | | 34G>T | | | | 20 | | | 0 | | | | 0 | | 20 | | | 0 | | | 0 | |
|  |  | G12V | | | 35G>T | | | | 10 | | | 0 | | | | 0 | | 10 | | | 1 | | | 10 | |
|  |  | G12D | | | 35G>A | | | | 8 | | | 0 | | | | 0 | | 8 | | | 1 | | | 12.5 | |
|  |  | G12A | | | 35G>C | | | | 3 | | | 0 | | | | 0 | | 3 | | | 0 | | | 0 | |
|  |  | G12F | | | 34_35GG>TT | | | | 2 | | | 0 | | | | 0 | | 2 | | | 1 | | | 50 | |
|  | |  | | |  | | | |  | | |  | | | |  | |  | | |  | | |  | |
| Codon 13 | | G13F | | | 37_38GG>TT | | | | 1 | | | 0 | | | | 0 | | 1 | | | 0 | | | 0 | |
|  | |  | | |  | | | |  | | |  | | | |  | |  | | |  | | |  | |
| Codon 61 | | Q61H | | | 183A>C, 183A>T | | | | 4 | | | 0 | | | | 0 | | 4 | | | 0 | | | 0 | |
|  |  | Q61L | | | 182A>T | | | | 1 | | | 0 | | | | 0 | | 1 | | | 0 | | | 0 | |
|  | |  | | |  | | | |  | | |  | | | |  | |  | | |  | | |  | |
| Rearrangement | | Amplification | | |  | | | | 4 | | | 0 | | | | 0 | | 4 | | | 0 | | | 0 | |
|  | |  | | |  | | | |  | | |  | | | |  | |  | | |  | | |  | |
| VUS | | D119H | | | 355G>C | | | | 0 | | | 1 | | | | 0 | | 1 | | | 0 | | | 0 | |
|  |  | D132H | | | 394G>C | | | | 0 | | | 1 | | | | 0 | | 1 | | | 0 | | | 0 | |
|  |  | E107K | | | 319G>A | | | | 0 | | | 1 | | | | 0 | | 1 | | | 0 | | | 0 | |
|  |  | E143K | | | 427G>A | | | | 0 | | | 1 | | | | 0 | | 1 | | | 0 | | | 0 | |
|  | |  |  | | |  | | | |  | | |  | |  | | | |  | | |  | | |  |
|  | |  |  | | |  | | | |  | | |  | |  | | | |  | | |  | | |  |
|  | |  |  | | |  | | | |  | | |  | |  | | | |  | | |  | | |  |
|  | |  |  | | |  | | | |  | | |  | |  | | | |  | | |  | | |  |
|  | |  |  | | |  | | | |  | | |  | |  | | | |  | | |  | | |  |
|  | |  |  | | |  | | | |  | | |  | |  | | | |  | | |  | | |  |
|  | |  |  | | |  | | | |  | | |  | |  | | | |  | | |  | | |  |
|  | |  |  | | |  | | | |  | | |  | |  | | | |  | | |  | | |  |
|  | |  |  | | |  | | | |  | | |  | |  | | | |  | | |  | | |  |
|  | |  |  | | |  | | | |  | | |  | |  | | | |  | | |  | | |  |
|  | |  |  | | |  | | | |  | | |  | |  | | | |  | | |  | | |  |
|  | |  |  | | |  | | | |  | | |  | |  | | | |  | | |  | | |  |
| ***ERBB2*** | |  |  | | |  | | | |  | | |  | |  | | | |  | | |  | | |  |
| **Alteration** | | **Protein change** | **Coding DNA sequence change** | | | | | | | **Known pathogenic** | | | **Likely pathogenic** | | **VUS** | | | | **Total n:o of alterations** | | | **No. of alterations in never smokers** | | | **% of alterations in never-smokers** |
| Exon 20 insertion | | A775_G776  insYVMA | 2324_2325ins  ATACGTGATGGC | | | | | | | 4 | | | 0 | | 0 | | | | 4 | | | 4 | | | 100 |
|  | |  |  | | |  | | | |  | | |  | |  | | | |  | | |  | | |  |
| Rearrangements | | Amplification |  | | |  | | | | 2 | | | 0 | | 0 | | | | 2 | | | 0 | | | 0 |
|  | |  |  | | | | | | |  | | |  | |  | | | |  | | |  | | |  |
| Exon 8 SNV | | S310F | 929C>T | | | | | | | 1 | | | 0 | | 0 | | | | 1 | | | 0 | | | 0 |
|  | |  |  | | | | | | |  | | |  | |  | | | |  | | |  | | |  |
| Exon 19 SNV | | D769H | 2305G>C | | | | | | | 1 | | | 0 | | 0 | | | | 1 | | | 0 | | | 0 |
|  | |  |  | | |  | | | |  | | |  | |  | | | |  | | |  | | |  |
| VUS | | A440T | 1318G>A | | | | | | | 0 | | | 0 | | 1 | | | | 1 | | | 0 | | | 0 |
|  |  | D880fs*24 | 2640_2642TGG>A | | | | | | | 0 | | | 0 | | 1 | | | | 1 | | | 0 | | | 0 |
|  |  | R188C | 562C>T | | | | | | | 0 | | | 0 | | 1 | | | | 1 | | | 1 | | | 100 |
|  |  | R929Q | 2786G>A | | | | | | | 0 | | | 0 | | 1 | | | | 1 | | | 0 | | | 0 |
|  |  | Splice site 3160-1G>A | 3160-1G>A | | | | | | | 0 | | | 0 | | 1 | | | | 1 | | | 0 | | | 0 |
|  | |  |  | | |  | | | |  | | |  | |  | | | |  | | |  | | |  |
|  | |  |  | | |  | | | |  | | |  | |  | | | |  | | |  | | |  |
|  | |  |  | | |  | | | |  | | |  | |  | | | |  | | |  | | |  |
|  | |  |  | | |  | | | |  | | |  | |  | | | |  | | |  | | |  |
|  | |  |  | | |  | | | |  | | |  | |  | | | |  | | |  | | |  |
|  | |  |  | | |  | | | |  | | |  | |  | | | |  | | |  | | |  |
|  | |  |  | | |  | | | |  | | |  | |  | | | |  | | |  | | |  |
|  | |  |  | | |  | | | |  | | |  | |  | | | |  | | |  | | |  |
|  | |  |  | | |  | | | |  | | |  | |  | | | |  | | |  | | |  |
|  | |  |  | | |  | | | |  | | |  | |  | | | |  | | |  | | |  |
|  | |  |  | | |  | | | |  | | |  | |  | | | |  | | |  | | |  |
|  | |  |  | | |  | | | |  | | |  | |  | | | |  | | |  | | |  |
|  | |  |  | | |  | | | |  | | |  | |  | | | |  | | |  | | |  |
|  | |  |  | | |  | | | |  | | |  | |  | | | |  | | |  | | |  |
|  | |  |  | | |  | | | |  | | |  | |  | | | |  | | |  | | |  |
|  | |  |  | | |  | | | |  | | |  | |  | | | |  | | |  | | |  |
| ***BRAF*** | |  |  | | |  | | | |  | | |  | |  | | | |  | | |  | | |  |
| **Alteration** | | **Protein change** | **Coding DNA sequence change** | | | | | | | **Known pathogenic** | | | **Likely pathogenic** | | **VUS** | | | | **Total n:o of alterations** | | | **No. of alterations in never smokers** | | | **% of alterations in never-smokers** |
| Class II | | G469V | 1406G>T | | | | | | | 5 | | | 0 | | 0 | | | | 5 | | | 0 | | | 0 |
|  | |  |  | | | | | | |  | | |  | |  | | | |  | | |  | | |  |
| Class III | | G466A | 1397G>C | | | | | | | 1 | | | 0 | | 0 | | | | 1 | | | 0 | | | 0 |
|  | |  |  | | | | | | |  | | |  | |  | | | |  | | |  | | |  |
| Others | | G464V | 1391G>T | | | | | | | 1 | | | 0 | | 0 | | | | 1 | | | 0 | | | 0 |
|  |  | G596R | 1786G>C | | | | | | | 1 | | | 0 | | 0 | | | | 1 | | | 0 | | | 0 |
|  |  | N581S | 1742A>G | | | | | | | 1 | | | 0 | | 0 | | | | 1 | | | 0 | | | 0 |
|  |  | S605C | 1813A>T | | | | | | | 1 | | | 0 | | 0 | | | | 1 | | | 0 | | | 0 |
|  |  | splice site 1178-2A>T | 1178-2A>T | | | | | | | 1 | | | 0 | | 0 | | | | 1 | | | 0 | | | 0 |
|  | |  |  | | |  | | | |  | | |  | |  | | | |  | | |  | | |  |
| VUS | | Q98R | 293A>G | | | | | | | 0 | | | 0 | | 1 | | | | 1 | | | 0 | | | 0 |
|  |  | T142S | 293A>G | | | | | | | 0 | | | 0 | | 1 | | | | 1 | | | 0 | | | 0 |
|  | |  |  | | |  | | | |  | | |  | |  | | | |  | | |  | | |  |
|  | |  |  | | |  | | | |  | | |  | |  | | | |  | | |  | | |  |
|  | |  |  | | |  | | | |  | | |  | |  | | | |  | | |  | | |  |
|  | |  |  | | |  | | | |  | | |  | |  | | | |  | | |  | | |  |
|  | |  |  | | |  | | | |  | | |  | |  | | | |  | | |  | | |  |
|  | |  |  | | |  | | | |  | | |  | |  | | | |  | | |  | | |  |
|  | |  |  | | |  | | | |  | | |  | |  | | | |  | | |  | | |  |
|  | |  |  | | |  | | | |  | | |  | |  | | | |  | | |  | | |  |
|  | |  |  | | |  | | | |  | | |  | |  | | | |  | | |  | | |  |
|  | |  |  | | |  | | | |  | | |  | |  | | | |  | | |  | | |  |
|  | |  |  | | |  | | | |  | | |  | |  | | | |  | | |  | | |  |
|  | |  |  | | |  | | | |  | | |  | |  | | | |  | | |  | | |  |
|  | |  |  | | |  | | | |  | | |  | |  | | | |  | | |  | | |  |
|  | |  |  | | |  | | | |  | | |  | |  | | | |  | | |  | | |  |
|  | |  |  | | |  | | | |  | | |  | |  | | | |  | | |  | | |  |
|  |  | | |  | | |  |  | | |  | | |  | | |  | | |  | | |  |  |  |

| ***BRCA1*** |  |  |  |  |  |  |  |  |  |
| --- | --- | --- | --- | --- | --- | --- | --- | --- | --- |
| **Alteration** | **Protein change** | **Coding DNA sequence change** | | **Known pathogenic** | **Likely pathogenic** | **VUS** | **Total n:o of alterations** | **No. of alterations in never smokers** | **% of alterations in never-smokers** |
| Rearrangement | Deletion |  |  | 0 | 1 | 0 | 1 | 0 | 0 |
|  |  |  | |  |  |  |  |  |  |
| VUS | E1001D | 3003A>T | | 0 | 0 | 1 | 1 | 0 | 0 |
|  | E1419K | 4255G>A | | 0 | 0 | 1 | 1 | 0 | 0 |
|  | A1308V | 3923C>T | | 0 | 0 | 1 | 1 | 0 | 0 |
|  |  |  |  |  |  |  |  |  |  |
|  |  |  |  |  |  |  |  |  |  |
|  |  |  |  |  |  |  |  |  |  |
| ***BRCA2*** |  |  | |  |  |  |  |  |  |
| **Alteration** | **Protein change** |  |  | **Known pathogenic** | **Likely pathogenic** | **VUS** | **Total n:o of alterations** | **No. of alterations in never smokers** | **% of alterations in never-smokers** |
|  | R3052Q | 9155G>A | | 0 | 1 | 0 | 1 | 0 | 0 |
|  | E409* | 1225G>T | | 1 | 0 | 0 | 1 | 0 | 0 |
|  | splice site 8331+2T>C | 8331+2T>C | | 1 | 0 | 0 | 1 | 0 | 0 |
|  |  |  |  |  |  |  |  |  |  |
| VUS | G2274V | 6821G>T | | 0 | 0 | 2 | 2 | 0 | 0 |
|  | L3101P | 9302T>C | | 0 | 0 | 1 | 1 | 1 | 100 |
|  | N1742I | 5225A>T | | 0 | 0 | 1 | 1 | 1 | 100 |
|  | T2007N | 6020C>A | | 0 | 0 | 1 | 1 | 1 | 100 |
|  |  |  |  |  |  |  |  |  |  |
|  |  |  |  |  |  |  |  |  |  |
|  |  |  |  |  |  |  |  |  |  |
|  |  |  |  |  |  |  |  |  |  |
|  |  |  |  |  |  |  |  |  |  |
|  |  |  |  |  |  |  |  |  |  |
|  |  |  |  |  |  |  |  |  |  |
|  |  |  |  |  |  |  |  |  |  |
|  |  |  |  |  |  |  |  |  |  |
| ***MET*** |  |  |  |  |  |  |  |  |  |
| **Alteration** | **Protein change** |  | | **Known pathogenic** | **Likely pathogenic** | **VUS** | **Total n:o of alterations** | **No. of alterations in never smokers** | **% of alterations in never-smokers** |
| Rearrangement | Amplification |  |  | 3 | 0 | 0 | 3 | 0 | 0 |
|  |  |  |  |  |  |  |  |  |  |
| Exon 14  alterations | Y1003C | 3008A>G | | 0 | 1 | 0 | 1 | 1 | 100 |
|  | Y1003* | 3009_3015CCGAGCT>G | | 1 | 0 | 0 | 1 | 0 | 0 |
|  | splice site 2888-21_2888-2del20 | 2888-21_2888-2del20 | | 0 | 1 | 0 | 1 | 0 | 0 |
|  | splice site 2888-26_2896del35 | 2888-26_2896del35 | | 0 | 1 | 0 | 1 | 0 | 0 |
|  | splice site 3028+3A>G | 3028+3A>G | | 0 | 1 | 0 | 1 | 0 | 0 |
|  | D1010H | 3028G>C | | 1 | 0 | 0 | 1 | 0 | 0 |
|  |  |  |  |  |  |  |  |  |  |
| VUS | T611I | 1832C>T | | 0 | 0 | 1 | 1 | 0 | 0 |
|  | V136I | 406G>A | | 0 | 0 | 1 | 1 | 0 | 0 |
|  | E1127K | 3379G>A | | 0 | 0 | 1 | 1 | 1 | 100 |
|  | I247V | 739A>G | | 0 | 0 | 1 | 1 | 0 | 0 |
|  | D1002_P1008del | 3006_3027>G | | 0 | 0 | 1 | 1 | 1 | 100 |
|  |  |  |  |  |  |  |  |  |  |
|  |  |  |  |  |  |  |  |  |  |
|  |  |  |  |  |  |  |  |  |  |
|  |  |  |  |  |  |  |  |  |  |
|  |  |  |  |  |  |  |  |  |  |
|  |  |  |  |  |  |  |  |  |  |
|  |  |  |  |  |  |  |  |  |  |
|  |  |  |  |  |  |  |  |  |  |
|  |  |  |  |  |  |  |  |  |  |
|  |  |  |  |  |  |  |  |  |  |
|  |  |  |  |  |  |  |  |  |  |
| ***SMARCA4*** |  |  |  |  |  |  |  |  |  |
| **Alteration** | **Protein change** |  |  | **Known pathogenic** | **Likely pathogenic** | **VUS** | **Total n:o of alterations** | **No. of alterations in never smokers** | **Percentage of alterations in**  **never smokers** |
|  | E1579* | 4735G>T | | 1 | 0 | 0 | 1 | 0 | 0 |
|  | E1212* | 3634G>T | | 0 | 1 | 0 | 1 | 0 | 0 |
|  | E512* | 1534G>T | | 0 | 1 | 0 | 1 | 0 | 0 |
|  | T910M | 2729C>T | | 1 | 0 | 0 | 1 | 0 | 0 |
|  | K1566_E1567>N* | 4698_4699GG>TT | | 0 | 1 | 0 | 1 | 0 | 0 |
|  | D1188Y | 3562G>T | | 0 | 1 | 0 | 1 | 0 | 0 |
|  | H1181Y | 3541C>T | | 0 | 1 | 0 | 1 | 1 | 100 |
|  | R1243L | 3728G>T | | 0 | 1 | 0 | 1 | 0 | 0 |
|  |  |  | |  |  |  |  |  |  |
| Rearrangement | Partial deletion |  |  | 0 | 1 | 0 | 1 | 0 | 0 |
|  |  |  |  |  |  |  |  |  |  |
| VUS | E1578D | 4734G>T VUS | | 0 | 0 | 1 | 1 | 0 | 0 |
|  | G33C | 96_97GG>TT VUS | | 0 | 0 | 1 | 1 | 0 | 0 |
|  | R1405Q | 4214G>A | | 0 | 0 | 1 | 1 | 1 | 100 |
|  |  |  |  |  |  |  |  |  |  |
|  |  |  |  |  |  |  |  |  |  |
|  |  |  |  |  |  |  |  |  |  |
|  |  |  |  |  |  |  |  |  |  |
|  |  |  |  |  |  |  |  |  |  |
|  |  |  |  |  |  |  |  |  |  |
|  |  |  |  |  |  |  |  |  |  |
|  |  |  |  |  |  |  |  |  |  |
|  |  |  |  |  |  |  |  |  |  |
|  |  |  |  |  |  |  |  |  |  |
|  |  |  |  |  |  |  |  |  |  |
