# Supplementary material for: Comprehensive genomic profiling of Finnish lung adenocarcinoma cohort reveals high clinical actionability and SMARCA4 altered tumors with variable histology and poor prognosis
Source: Neoplasia. 2022 Aug 11;32:100832. doi: 10.1016/j.neo.2022.100832 (PMC9391575; doi:10.1016/j.neo.2022.100832)
Supplement: Supplementary file 3 [file mmc3.docx]

**Supplementary Table 3.** Genes in which the frequency of any alterations vary between never-smokers (n=30, indicated with green) and smokers (n=104, indicated with orange) (p<0.05).

| **Gene** | **No. of never-smokers (%)** | **No. of smokers (%)** |
| --- | --- | --- |
| *EGFR* | 15 (50.0) | 17 (16.3) |
| *MDM2* | 7 (23.3) | 3 (2.9) |
| *FRS2* | 7 (23.3) | 4 (3.8) |
| *CDKN2B* | 7 (23.3) | 8 (7.7) |
| *NBN* | 0 (0) | 13 (12.5) |
| *PTPRD* | 0 (0) | 13 (12.5) |
| *EPHA5* | 0 (0) | 13 (12.5) |
| *GRIN2A* | 0 (0) | 14 (13.5) |
| *HGF* | 0 (0) | 14 (13.5) |
| *EPHA3* | 0 (0) | 17 (16.3) |
| *MLL2* | 1 (3.3) | 19 (18.3) |
| *KEAP1* | 0 (0) | 19 (18.3) |
| *RUNX1T1* | 0 (0) | 21 (20.2) |
| *ARID1B* | 1 (3.3) | 22 (21.2) |
| *PREX2* | 1 (3.3) | 23 (22.1) |
| *STK11* | 0 (0) | 27 (26.0) |
| *SPTA1* | 4 (13.3) | 34 (32.7) |
| *LRP1B* | 3 (10) | 35 (33.7) |
| *KRAS* | 2 (6.7) | 48 (46.2) |
| *TP53* | 9 (30) | 64 (61.5) |
