# Supplementary material for: Comprehensive genomic profiling of Finnish lung adenocarcinoma cohort reveals high clinical actionability and SMARCA4 altered tumors with variable histology and poor prognosis
Source: Neoplasia. 2022 Aug 11;32:100832. doi: 10.1016/j.neo.2022.100832 (PMC9391575; doi:10.1016/j.neo.2022.100832)
Supplement: Supplementary file 4 [file mmc4.docx]

| **Patient** | **Predominant pattern** | ***SMARCA4***  **variant** | **Type of**  **variant** | **SMARCA4**  **expression (IHC)** | **TTF-1 expression**  **(IHC)** | **Stage** | **TMB value**  **(muts/MB)** | **Survival (years)** |
| --- | --- | --- | --- | --- | --- | --- | --- | --- |
| Patient a | Acinar | T910M | Missense | diffusely positive | diffusely positive | 2 | 4,39 | 3,0 |
| Patient b | Acinar | H1181Y | Missense | diffusely positive | diffusely positive | 1 | 10,53 | 1,9 |
| Patient c | Acinar | - | Partial deletion | diffusely positive | diffusely positive | 2 | 2,63 | 3,5 |
| Patient d | Acinar | R1243L | Missense | heterogenous | negative | 1 | 6,14 | 4,9 |
| Patient e | Acinar | G33C | Missense | heterogenous | diffusely positive | 1 | 88,63 | 10,4 |
| Patient f | Acinar | R1405Q | Missense | diffusely positive | diffusely positive | 3 | 1,75 | 2,6 |
| Patient g | Papillary | E1212* | Truncating | diffusely positive | diffusely positive | 1 | 2,63 | 2,8 |
|  |  | D1188Y | Missense |  |  |  |  |  |
| Patient h | Solid | E512* | Truncating | negative | diffusely positive | 3 | 16,67 | 0,9 |
| Patient i | Solid | E1579* | Truncating | negative and patchy positive areas | negative | 3 | 20,18 | 1,7 |
|  |  | E1578D | Missense |  |  |  |  |  |
| Patient j | Solid | K1566_E1567>N* | Truncating | negative (solid) and diffusely positive (lepidic) areas | N/A | 3 | 6,14 | 0,7 |

**Supplementary Table 4.** The clinicopathological characteristics of the individual tumors with *SMARCA4* alterations.
